# Commentary to “The human intelligence evolved from proximal *cis*‐regulatory saltations”

**DOI:** 10.1002/qub2.92

**Published:** 2025-01-14

**Authors:** Lei M. Li

**Affiliations:** ^1^ Academy of Mathematics and Systems Science Chinese Academy of Sciences Beijing China; ^2^ School of Mathematical Sciences University of the Chinese Academy of Sciences Beijing China

**Keywords:** David Hume, entropy, Gauguin, human intelligence, saltation

In this rapidly evolving era of artificial intelligence, we are still lacking the knowledge of human natural intelligence. The article [1] reported that the saltations in the proximal *cis*‐regulatory modules could, to some extent, explain the miraculous human intelligence at the molecular level. To help readers understand the work, I made some comments from both historical and methodological perspectives.

## 1 HISTORICAL COMMENTS

### 1.1 The fundamental questions: “Where do we come from? What are we? Where are we going?”

The questions are the folk version of the scientific one we aimed to address in our work [1]. They were the title of the famous painting created by Paul Gauguin in Tahiti in 1897 [2]. Gauguin attended the Catholic boarding school Petit Séminaire de La Chapelle‐Saint‐Mesmin during 1859–1862 and 1863–1864. The teacher of his Catholic liturgy class was the Bishop of Orléans, Félix‐Antoine‐Philibert Dupanloup. In his catechism, Dupanloup posed “Where does humanity come from?”, “Where is it going to?”, and “How does humanity proceed?”. Gauguin was thought to be influenced by Dupanloup’s version of fundamental questions. Tracing back further, it is very likely that Dupanloup came to the questions after knowing about Darwin’s theory and his book *On the Origin of Species*, which was published in 1859.

### 1.2 David Hume’ account of human nature

The work by David Hume made a central influence on the theory of evolution, as Charles Darwin acknowledged. Hume published *A Treatise of Human Nature* [3] in 1739–1740 anonymously and *Enquiries concerning Human Understanding* [4] in 1748. He demystified human nature, including intelligence as the major component, by decomposing it into a minimal set of motif machinery, as Isaac Newton did in physics. For example, he broke down perceptions into two categories: impressions and ideas, either simple or complex. Hume took an empirical approach to the decomposition. That is, every motif was warranted by experience, primarily by his own experience along with observations on others’. We adopted a similar empirical and reductionist approach except that the experience is replaced by high quality and annotated genomes, which are the genetic and molecular basis of humans.

### 1.3 “Nothing in biology makes sense except in the light of evolution”

Professor Dobzhansky’s statement as shown in Ref. [5] serves as a guiding principle for our research design. In the case of humans, fortunately, we have several extant relative species as shown in Figure 1 in Ref. [1]. Their complete genomes form the basis for the comparative studies. Even though the number of genomes is small, it suffices, as explained in Ref. [1], to identify the two saltations between apes and humans. Notably, the surprising saltations were discovered solely from proximal regulatory sequences around transcription start sites. Apart from the small difference between protein sequences of humans and apes as explained in Ref. [1], we next make an entropy‐based argument to emphasize the importance of the regulatory sequences.

## 2 METHODOLOGICAL COMMENTS

### 2.1 Transcription from DNA to RNA reduces information entropy

We argue, in molecular biology, the transcriptional machinery that copies a segment of DNA into RNAs, is a major step where the entropy of genetic information undergoes reduction. We illustrate this by an example. Suppose we have eight protein genes, each of which has a distinct DNA sequence in a genome. Since every cell of the same species shares the same genome, the chance of picking one gene randomly from genomes of many cells would be 1/8=0.125. The entropy of such a probability distribution is therefore $-\sum_{k=1}^{8}0.125\times \log_{2}\left(0.125\right)=3\;bits$. This uniform distribution reaches the maximum entropy. In a specific cell under a specific stage, the eight genes are not uniformly transcribed into mRNAs. Suppose the first gene generates 1024 copies, the second and third genes generate 512 copies each, and the remaining genes are not transcribed at all. Then, after normalizing the counts, the distribution of the eight gene transcripts becomes 0.5, 0.25, 0.25, 0, 0, 0, 0, and 0. Its entropy is $-0.5\times \log_{2}\left(0.5\right)-2\times 0.25\times \log_{2}\left(0.25\right)=1.5\;\text{bits}$. Thus, a reduction of 1.5 bits in entropy is achieved through the process of transcription.

After RNAs are transcribed from DNA, the situations are more complex. Mechanisms such as alternative splicing and RNA editing can increase entropy. During translation, if we assume that one copy of mRNA would lead to a constant number of proteins, entropy would remain unchanged. However, variations in mRNA lifetimes, translational efficiency, and cell conditions will alter entropy in one way or another. In contrast, DNA replication makes uniform copies of the entire genome without altering entropy, provided no errors occur. From this information perspective, gene transcription from DNA to RNA is a major step that reduces entropy in cells. Of note, the aforementioned reduction of entropy is understood in the framework of information theory rather than statistical mechanics, as mentioned in Schrödinger’s argument [6].

### 2.2 Transcription capacity

How is the entropy reduction realized in transcription? The transcription machinery is like a printing factory, whose production is controlled by a complex regulatory program. The single cell work indicates that the number of transcription factor bindings not only controls the number of mRNAs synthesized at individual gene loci [7] but also contributes to promoter activation [8]. The maximum number of bindings is limited by the number of partner *cis*‐regulatory elements, which we termed as transcription capacity [9]. The *cis*‐regulatory element frequency (CREF) matrix measures a species’ transcription capacity written in its DNA rather than specific expression profiles, which are subject to cell types and conditions. The holistic picture of *cis*‐element multiplicities shown in Figure 3 in Ref. [1] demonstrates their diversity across different genes.

### 2.3 Dual eigen‐analysis

Deciphering the *cis*‐regulatory element frequency matrix is essentially a dimension reduction problem. A standard tool in textbooks is the principal component analysis (PCA), which is the eigen decomposition of the sample covariance matrix. For example, the covariance of a data matrix of 4 variables and 10,000 observations is simply a 4 by 4 square matrix. This transformation from the original data matrix to covariance was appealing before computers became available. In the case of a *cis*‐regulatory element frequency matrix, which typically consists of about 20,000 rows (genes) and 1400 columns (motifs), we would rather carry out singular value decomposition directly on the data matrix. The baseline component roughly corresponds to adjusting both row and column averages. By contrast, in PCA, its first step of calculating the covariance matrix is subtracting each entry by its column average.

### 2.4 Eigen postulate in quantum mechanics

The singular value decomposition stratifies the interaction strengths between *cis*‐element and gene pairs into discrete and orthogonal eigenvectors. In each pair of polarized eigenvectors, the *cis*‐regulators and their gene targets are regrouped by weights at the poles. Although the correspondence well aligns with known examples, the underlying mechanism by which eigenvectors operate in cells remains an open question. While further investigations are needed to bridge the gap between theories and experimental data, the analogy in quantum mechanics might be relevant for understanding the situation.

Quantum mechanics, one cornerstone of current physics, requires a solid mathematical model to explain the observations in various experiments. Over the past century, several equivalent theoretical frameworks have been developed alongside the formulation of axioms within a mathematical context [10]. One postulate in the axioms is the following: Every measurable physical quantity A in an isolated physical system, whose state is represented by a vector in Hilbert space H, is described by a Hermitian operator A acting in H; a measurement of A must be one eigenvalue of the corresponding A. So far the eigen‐postulate has been accepted for its compatibility with experimental data.

### 2.5 Selection of *cis*‐elements

In this study, the motifs of *cis*‐regulatory elements were from the long‐standing database TRANSFAC. Notably, the saltations observed in the 4th and 9th CREF modules are primarily characterized by cellular processes related to brain functions. Assessing the sensitivity of these results to the choice of *cis*‐elements is crucial, as it raises the question of whether the emergence of intelligence modules in humans could result from a possible weighting bias toward brain‐specific *cis*‐elements. We addressed this possibility with several observations. First, found at the two poles of the 4th and 9th motif eigenvectors, most *cis*‐elements that play important regulatory roles in the brain cells, do so in other tissues as well. For example, SP1, whose binding motifs SP1_03, SP1_Q2_01, SP1_02, and SP1_Q4_01 rank in the top 100 of the 4th motif eigenvector, regulates neuronal differentiation and neuronal survival through interactions with other factors such as MAZ, SP3, *c.f.* Figure 4 in Ref. [9]. Nevertheless, SP1 is a general regulator across various cell types rather than exclusive to brain cells. We searched the top 100 motifs at each pole of the 4th eigenvector for those corresponding to brain‐specific transcription factors. According to the annotations of UniProt [11], only ZIC1, ZIC2, and ZIC3 possess both brain‐specific regulatory functions and brain‐enriched expressions, and their binding motifs account for only 5 out of 200. Second, the modules at the 4th and 9th levels include not only molecular processes in brain cells but also others such as the development of cochlea development, which has received limited attention so far in the literature. Third, in the simulation study reported earlier [9], we repeated the CREF analysis by randomly sampling 80% of the *cis*‐elements available from the database. The distribution of the singular values as shown in Figure 2C in Ref. [9] indicates that the 1st, 2nd, 3rd, and 6th singular values were well separated from their neighbors, while the 4th and the 5th overlap to some extent. This distribution of singular values, alongside the theoretical and empirical perturbation analysis of eigenspace as shown in Refs. [1, 9], reinforces the significance of the saltations observed between the 4th and the 5th levels.

Next, we elaborate on the intelligence traits found from the saltations.

## 3 HUMAN INTELLIGENCE

### 3.1 Long‐term memory

The CREF analysis revealed at least four molecular evidences to support the enhanced long‐term memory of humans. First, “regulation of synapsis plasticity” is among the most significantly enriched biological processes at the pole of the 4th human gene‐eigenvector. Synapsis plasticity was thought to be a fundamental mechanism contributing to memory storage [12]. Second, numerous genes involved in the Schaffer collateral–CA1 synapse were ranked among the top 1500 in the 9th human gene‐eigenvector. Schaffer collateral is a critical pathway for activity‐dependent plasticity and the dynamic process of memory development in the hippocampus. Third, the biological process “regulation of oligodendrocyte differentiation” is significantly enriched at the pole of the 4th gene‐eigenvector and so is “myelin maintenance” at the pole of the 9th gene‐eigenvector. In recent years, new studies have shown that the activity‐dependent formation of myelin contributes to memory consolidation and recall [13, 14]. Myelinated axons are the major components of white matter. It was reported that the prefrontal white matter volume is disproportionately larger in humans than in other primates [15]. Recently, we reported that compared to mice, dogs are more similar to humans in their high level and prolonged expression of myelin proteins in the prefrontal cortex and hippocampus, and the expression patterns were in line with the CREF module analysis [16]. Thus, the CREF modules explained the similar social and cognitive abilities between humans and dogs at the molecular level. Fourth, the protein CEBPG, a transcription factor, can form stable heterodimers with the protein CEBPB which is a key regulator of synaptic plasticity and memory formation. CEBPG ranked high in the human 9th gene‐eigenvector, and its promoter region contains a human‐specific AluYa5 insertion. Together with various other evidences [1, 9], it is clear that long‐term memory is one of the most prominent intelligence humans acquired from the saltations.

If long‐term memory was a trait that was dramatically enhanced over a relatively short evolutionary timespan, it is then understandable that humans, upon gaining the ability, felt urged to ask themselves where our ancestors were from, yet found no definite answer beyond a time point. That is to say, the rapid evolution of long‐term memory might lead humans to a situation with enhanced capability and capacity of memory yet without much to remember about their ancestors’ history.

Returning to Hume’s account of the human mind, he distinguished two kinds of impressions: impressions of sensation and impressions of reflection. The former involves primarily feelings from our senses, whereas the later requires certain long‐term memory. In contrast, ideas are much less vivid, as he termed, and are “the faint images”, which obviously correspond to those stored in long‐term memory.

Humans’ long term memory could be formed by factors at different scales. From the perspective of society, Dawkins [17] proposed the notion of MEME as a kind of memory unit. A meme is an idea, behavior, or style that spreads through imitation from one person to another within a culture. It often carries symbolic meaning that represents a specific theme or phenomenon. In contrast, the discoveries made by the CREF module saltations suggest that humans’ long term memory has a molecular basis that apes do not possess. And so have humans’ abilities of language and music, which are major vehicles for the spread of memes.

### 3.2 Cochlea development

The ability of speaking oral language is without doubt one intelligence that humans have. During early development, a young child must first hear the sounds of others’ speech so as to learn to understand them and to speak themselves. Children with sensorineural hearing loss experience difficulties in developing the ability of producing intelligible speech. On the other hand, it was reported that cochlear implantation of congenitally deaf children at early ages helps spoken language development [18]. The mechanism is likely through dynamic gene expression, subject to epigenetic control. Over the course of evolution, the size, position, and orientation of the human cochlea underwent subtle changes. The CREF analysis demonstrated that cochlea development is a key biological process in the emerged 4th and 9th human gene‐eigenvectors, which are also linked to long‐term memory.

Other than language, music is a key component of the human civilizations. It is unclear whether language or music came into human life first. The evolution of music is manifested by the musical instruments across historical periods. The bone flutes found in Swabian Jura, Germany, dated back to at least 40,000 years ago [19]. The advance of musical instruments reflects the evolution of human hearing ability.

Despite its significance in both language and music, insufficient attention has been given to the cochlea and inner ear development in the research of human evolution and intelligence.

### 3.3 Social behavior, society, and collective intelligence

Along the 4th human gene‐eigenvectors, quite a portion of genes are annotated to be social behavior, adult behavior, and regulation of behaviors. Notable among them are genes MBD5, GRP, SHANK1, SHANK3, NRXN1, NRXN2, and NLGN2. The result indicates that social behavior is an effect of natural selection. Before the rotated human’s motif‐ and gene‐eigenvectors were revealed to us, it is my belief that our social behavior was shaped and cultivated by tradition and education. For example, in the Chinese culture that I was raised, the teaching “the spirit of benevolence is to exercise self‐discipline and return to ritual” (克己复礼为仁) was documented in the classic book *Analects*, dating back to at latest 200 BC. The new discovery indicates that the genes relating to social behavior are expressed more in humans through transcriptional regulation, implying certain portion of social behaviors are already encoded in our current genome.

The rational social behavior allowed humans to establish societies of law, commence, academia etc. Through societies, collective intelligence emerged and were accumulated over time. Take academia for example, Pierre de Fermat formulated his conjecture, known as Fermat’s Last Theorem, around 1637. Its validity remained unresolved until 358 years later, Andrew Wiles published the first successful proof in 1995 by putting together several mathematical theories. This collaborative effort across the globe has significantly enhanced human mathematical intelligence.

### 3.4 Learning ability

The enhanced learning abilities revealed by the rotated 4th and 9th gene‐eigenvectors form the foundation of human intelligence. Among them, associative learning is most sophisticated. Hume was the first to categorize the three types of associations: resemblance, contiguity in time and place, and from cause to effect. All of them rely on not only long‐term memory but also certain computations such as Boolean operations and sorting.

### 3.5 Modular intelligence

In contrast to a protein gene, which is usually associated with a specific trait, the saltation of a CREF module regroups a large number of genes at the transcription level. If the genes within a module could indeed be co‐transcribed during development, then the combination of different traits may arise. For example, absolute pitch, which is the ability to identify or produce a musical note without any external reference, is the combination of two traits: the ability of recognizing the pitch of a note and the retention of an internal reference in long‐term memory. Although it is rare, only about 1 in 10,000 people as widely reported without sufficient evidence [20], absolute pitch has become an existence in humans.

The final remark is that the CREF regulatory modules, though still in a primitive and heuristic form, is the first simple theory capable of explaining saltation of certain human traits, particularly those related to intelligence.

## AUTHOR CONTRIBUTIONS

**Lei M. Li**: Conceptualization; writing ‐ original draft; writing ‐ review & editing.

## CONFLICT OF INTEREST STATEMENT

The author declares that he has no conflict of interest.

## Data Availability

All data used in the commentary are included in the main article and its supplementary materials file.
